# Genetic and Systematic Approaches Toward G Protein-Coupled Abiotic Stress Signaling in Plants

**DOI:** 10.3389/fpls.2018.01378

**Published:** 2018-09-20

**Authors:** Ting-Ying Wu, Daisuke Urano

**Affiliations:** ^1^Temasek Life Sciences Laboratory, Singapore, Singapore; ^2^Department of Biological Sciences, National University of Singapore, Singapore, Singapore

**Keywords:** systems biology, bioinformatics, G proteins, environmental stress, omics

## Abstract

Heterotrimeric G protein, composed of Gα, Gβ, and Gγ subunits, modulates plant adaptations to environmental stresses such as high salinity, drought, extreme temperatures and high light intensity. Most of these evidence were however derived solely from conventional genetics methods with which stress-associated phenotypes were compared between wild type and various G protein mutant plants. Recent advances in systematic approaches, mainly transcriptome and proteome, have contributed to in-depth understanding of molecular linkages between G proteins and environmental changes. Here, we update our knowledge on the roles of G proteins in abiotic stress responses. Furthermore, we highlight the current whole genome studies and integrated omics approach to better understand the fundamental G protein functions involved in abiotic stress responses. It is our purpose here to bridge the gap between molecular mechanisms in G protein science and stress biology and pave the way toward crop improvement researches in the future.

## Introduction to G proteins

Plants experience frequent changes in their growth environments which impede or alter their normal development. Environmental conditions include biotic stresses such as pathogen infection and abiotic stresses such as drought, high salinity, heat, cold, excessive light, high ultraviolet B (UVB) radiation, nutrient deficiency and accumulation of toxic metals in the soil. Due to the increased frequency of extreme weather and climate change in recent years, the adverse effects from those abiotic stresses have been accelerated in plants (Zhu, 2016). As a sessile organism, plants have developed many mechanisms to cope with unfavorable environments. How plants use the complicated combination of transcriptional and/or translational reprogramming to gain stress tolerance are pivotal biological questions. In this review, we will discuss the role of G protein genes in abiotic stress responses from the aspect of morphological adaptations to molecular mechanisms. We then further highlight the potential strategies to systematically integrate G protein science and stress biology.

**Figure 1:**
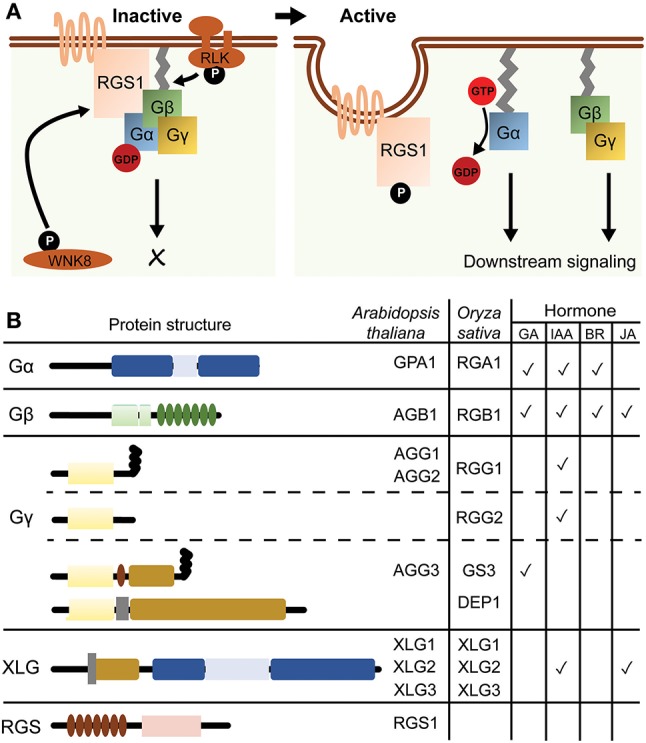
A model of G protein signaling pathway and their structural characteristics. **(A)** A model for G protein cycle in Arabidopsis. AtRGS1 keeps the Gα*βγ* complex in the inactive state by augmenting the hydrolysis of GTP to GDP. AtGRS1 is phosphorylated at the C terminus by membrane-associated or cytosolic kinases such as WNK8 and receptor-like kinases (RLK), and then undergoes endocytosis. Upon uncoupling from AtRGS1, Gα subunit is able to exchange GDP for GTP and is detached from Gβγ dimer, then activates the downstream effectors. **(B)** Domain architectures of Arabidopsis and rice G protein subunits and AtRGS1. AtGPA1 contains a Ras-like domain and a helical domain. AGB1 contains a coiled-coil domain in the N-terminal region and seven WD40 repeats. Gγ subunit can be classified into type A, B and C subgroups. Type A is composed of a Gγ-domain and a CaaX motif, while type B is composed of only a Gγ-domain. Type C contains a Gγ-domain, a cysteine-rich domain, a NLS, a CaaX motif and a transmembrane domain. Arabidopsis has no type B Gγ subunit. XLGs are composed of a NLS, a cysteine-rich domain, a Ras-like domain and a helical domain. AtRGS1 contains a 7-TM domain and a RGS domain. Rice has no RGS homolog. The table on the right summarizes G protein subunits related to hormone regulations, as discussed in the main text.

Heterotrimeric G protein, composed of Gα, Gβ, and Gγ subunits, is a well-conserved signaling protein that functions as a molecular switch in eukaryotes. In the steady state, Gα subunit holds a guanosine diphosphate (GDP) and forms an inactive complex with an obligate Gβγ dimer. Upon nucleotide exchange on Gα for a guanosine triphosphate (GTP), GTP-bound Gα dissociates from Gβγ then modulates the activity of downstream signaling proteins (Kaziro et al., 1991). While seven-transmembrane (7TM) G protein-coupled receptors (GPCRs) predominantly modulate the activity of heterotrimeric G protein in animals, single-transmembrane receptor kinases are the primary regulators of plant heterotrimeric G protein rather than hypothetical GPCR candidates with 7TM helices, such as G protein Coupled Receptor 1 (*GCR1*) (Aranda-Sicilia et al., 2015; Liang et al., 2016; Tunc-Ozdemir et al., 2016; Yu et al., 2018). In contrast to the receptor kinases, Regulator of G protein Signaling (RGS) negatively modulates the activity of G protein on the plasma membrane (Chen et al., 2003; Urano et al., 2015; Hackenberg et al., 2017; Figure 1A). Typical seed plants have two types of Gα; a canonical Gα and a non-canonical extra-large Gα (XLG), a single type of Gβ and three types of Gγ; Type-A, -B and -C Gγ subunits (Figure 1B).

The Arabidopsis genome contains one canonical Gα (*GPA1*), three XLG (*XLG1, XLG2* and *XLG3*), one Gβ (*AGB1*) and three Gγ (*AGG1, AGG2*, and *AGG3*) genes. Genetic ablation of some G protein genes confers various anomalous morphologies including leaf and flower shape, hypocotyl elongation and root mass and architecture. G protein mutations also alter the sensitivity to growth hormones including auxin, gibberellic acid (GA) and brassinosteroid (BR). In general, Gα mutants are hyposensitive to auxin, GA and BR, while Gβ mutants are hypersensitive to auxin but hyposensitive to GA and BR (Ueguchi-Tanaka et al., 2000; Ullah et al., 2001, 2003; Wang et al., 2006; Gao et al., 2008; Oki et al., 2009; Chakravorty et al., 2011). Some of these developmental phenotypes and hormonal responses are comparable in Gα, Gβ, or Gγ null mutants while others are opposite between these mutant lines. The complete knockout of *GPA1/XLG* or three types of *AGG* mimics all known phenotypes conferred by the null mutation in *AGB1* (Urano et al., 2016a) For further details of G protein functions in plant development and hormone perception, readers may refer to previous review articles (Assmann, 2004; Urano et al., 2013, 2016b; Pandey and Vijayakumar, 2018).

## G proteins and abiotic stress response in plants

Besides regulating several developmental processes and phytohormone responses, plant G proteins modulate a broad range of abiotic and biotic stress responses. Plants cope with abiotic stresses such as high salinity, drought, high light and extreme temperatures through the activation of dynamic signaling transductions in the cell. This section summarizes the relationship between G protein pathways and a variety of environmental changes.

### Salt stress

**Figure 2:**
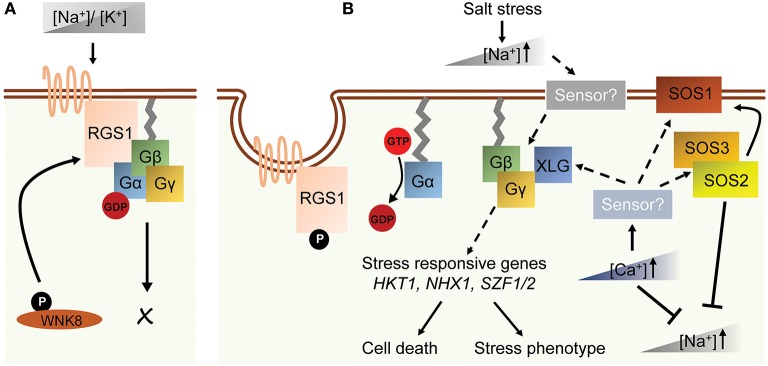
A schematic model of G protein-related salt response in Arabidopsis. **(A)** In normal growth condition, the homeostasis of [Na^+^] and [K^+^] is balanced. The G-protein complex is not activated. **(B)** Salt stress causes the elevation of cytosolic [Na^+^] concentration that is sensed by hypothetical sodium sensors on plasma membrane or in cytosol. Breakdown of ion homeostasis activates following signaling pathways. These include increasing of [Ca^2+^], activation of *SOS* genes and induction of Gβγ- and XLG-regulated genes such as *HKT1, SZF1/2* and *NHX1*. The subsequent signaling pathways then invoke stress responses and cell death in plants. Dashed arrows indicate indirect or hypothetical regulations, and solid arrows indicate direct regulations.

High soil salinity causes osmotic and ionic toxicity in plants resulting in reduced plant growth and crop yield (Zhu, 2002). High osmolarity rapidly inhibits cell proliferation in shoot apical meristem hence slowing down plant growth while ionic toxicity causes necrosis in the leaf tips and margins. Arabidopsis *AGB1*, triple *XLG*, and triple *AGG* null mutants exhibit smaller and chlorotic leaves when grown on NaCl-containing medium, whereas the seedlings and leaves of wild type plants remain greenish (Colaneri et al., 2014; Yu and Assmann, 2015; Liang et al., 2017). The hypersensitive phenotype of *agb1* is likely due to ionic toxicity, since *agb1* shows a similar leaf-bleaching phenotype with different ionic treatments but not with a changing water content (Colaneri et al., 2014; Yu and Assmann, 2015). Arabidopsis *agb1* mutant accumulates Na^+^ in both shoots and roots (Yu and Assmann, 2015, 2016), suggesting that AGB1 regulates Na^+^ fluxes in roots and the translocation of Na^+^ from roots to shoots (Yu and Assmann, 2015). Arabidopsis *gpa1* and *rgs1* mutants contrastingly show larger and less chlorotic leaves under NaCl treatment (Colaneri et al., 2014). In accord with the phenotypes in Arabidopsis, Gα-null mutation in rice and maize attenuated leaf senescence, chlorophyll degradation and cytoplasm electrolyte leakage caused by a high concentration of sodium chloride (Urano et al., 2014). Similarly, overexpression of *RGG1* in rice improved salt tolerance without affecting yield (Swain et al., 2017), suggesting a conserved stress-related role of heterotrimeric G protein across spermatophyte linages (Table 2). Several G protein interactors are genetically associated with G-proteins in the salt stress response. For example, a knockout mutant for With No Lysine 8 (WNK8) kinase, which phosphorylates RGS1 and induces its endocytosis, improved salt tolerance additively with *rgs1* in Arabidopsis (Urano et al., 2012, 2014; Colaneri et al., 2014; Cao-Pham et al., 2018). Recently, SALT INDUCIBLE ZINC FINGER 1, and 2 (SZF1 and SZF2) were found to be involved in salt stress response in the XLG-dependent pathway (Liang et al., 2017) Several Na+ transporters and sensors such as *SOS, HKT1*, and *NHX1* showed contradicted expression level in *agb1* mutants under salt stress (Figure 2; Ma et al., 2015a; Yu and Assmann, 2015). Therefore, the comprehensive molecular mechanism has not yet been deciphered (Table 1 and Figure 2).

**Table 1:** Response of Arabidopsis G protein mutants to environmental stresses and the stress hormone ABA.

**Mutants**	**Tolerance**	**Conditions**	**Stress phenotype**	**OMICS**	**References**
**SALT**					
*gpa1*	+	Hydroponic growing	Higher % of green seedlings	NA	Colaneri et al., 2014; Yu and Assmann, 2015; Liang et al., 2017
		Agar plates 50–250 mM NaCl			
*agb1*	– – –		Chlorotic seedlings		
			Reduces chlorophyll content		
			Reduces fresh weight		
			Lower survival rate		
			Lower stomata aperture size		
			Higher shoot ABA content		
			Higher Na+ accumulation		
*gpa1agb1*	+		Phenocopy *gpa1*		
*agg1, 2, 3*	– – –		Chlorotic seedlings		
			Lower % of green seedlings		
*xlg1, 2, 3*	– – –		Reduces plant size		
*gpa1xlg1, 2, 3*	– – –		Reduces green leaf area		
*rgs1*	++		Higher % of green seedlings		
*agb1rgs1*	– – –		Phenocopies *agb1*		
**DROUGHT**					
*gpa1*	+++	Dry soil20–40% soil water	Increases total Transpiration efficiency (TE) in both vegetative and bolting/ flowering stages	NA	Pandey and Assmann, 2004; Nilson and Assmann, 2010a,b
			Reduces TE in the inflorescence		
			Reduces stomata density		
			Reduces plant fitness		
			Increases plasticity for inflorescence		
*agb1*	– – –		Enhances fitness		
			Reduces plasticities in inflorescence height, fruit number and seed per fruit		
			Increases seed production		
*gpa1agb1*	– – –		Enhances fitness		
			Plasticities are similar to those in *agb1*, except for inflorescence height		
*gcr1*	++		Increases plasticity for fruit number Lower rate of water loss		
**OZONE**					
*gpa1*	+++	O_3_ controlled chamber500–700 ppb, 5–250 ppb	No leaf curvature	Transcriptome gpa1agb1, 125 ppb O_3_ treated for 3 h and 2 days.	Booker et al., 2004, 2012; Joo et al., 2005
			Chlorosis and necrotic lesions		
			WT level of net photosynthesis		
			Reduces cell death and ion leakage		
			Reduces ROS production		
*agb1*	– –		Reduces leaf curvature ratio		
			Severe chlorosis and necrotic lesions		
			WT level of net photosynthesis		
			Significantly lower chlorophyll concentration		
			Reduces leaf mass per leaf area		
			Induces cell death and ion leakage		
*gpa1agb1*	+++		Phenocopies gpa1		
*gcr1*	– – –		Reduces leaf curvature ratio		
			Severe chlorosis and necrotic lesions		
			WT level of net photosynthesis		
			Significantly lower chlorophyll concentration		
			Reduces leaf mass per leaf area		
*rgs1*	– – –		Reduces leaf curvature ratio		
			Severe chlorosis and necrotic lesions		
			WT level of net photosynthesis		
			Significantly lower chlorophyll concentration		
			Reduces leaf mass		
**UVB**					
*gpa1*	?	0.5 W m^−2^ UV-B	Increases stomatal aperture size	NA	Seo et al., 2004, 2007; Baker, 2008; Galvez-Valdivieso et al., 2009; He et al., 2013
			Reduces H_2_O_2_ production		
*cGPA1*	?		WT response to stomata and H_2_O_2_ production		
**HIGH LIGHT**					
*gpa1*		750 μmol m^−2^ s^−1^ PPFD	H_2_O_2_ production	NA	Seo et al., 2004, 2007; Baker, 2008; Galvez-Valdivieso et al., 2009; He et al., 2013
			APX2 gene expression		
*agb1*					
**ER STRESS**					
*gpa1*	+++	12.5–75 ng/ml	WT phenotype	NA	Wang et al., 2007; Chen and Brandizzi, 2012; Cho et al., 2015
	– – –	15–30 μg/ml			
*agb1*	– – –	Tunicamycin	Lower survival rate		
	+++		Lower fresh weight		
			Leaf senescence and damage		
			Smaller seedlings		
*gpa1agb1*	+++		WT phenotype		
*xlg1, 2, 3*	–		Lower survival rate		
*gpa1xlg1, 2, 3*	– –		Lower survival rate		
*agg2*	+++		WT phenotype		
*agg3*	+++		WT phenotype		
*agg1, 2, 3*	– – –		Lower survival rate		
**TEMPERATURE**					
agg2	?	29°C	Early flowering	NA	Thung et al., 2013
**ABA**					
*gpa1*	– –		Reduces primary root length		
			Slightly hypersensitive to ABA-induced inhibition of seed germination		
			Insensitive to ABA-activated Ca^2+^ current		
*agb1*	– – –		Reduced primary root length	Transcriptome	Wang et al., 2001; Pandey and Assmann, 2004; Pandey et al., 2006, 2010; Zhao et al., 2010; Alvarez et al., 2011; Jin et al., 2013
			Hypersensitive to ABA-induced inhibition of seed germination	Metabolome	
			ABA-related genes are highly upregulated	Proteome	
*gpa1agb1*	– – –	1–10 μM	Phenocopies *agb1*		
		ABA			
*xlg1, 2, 3*	+++		ABA-hyposensitive root phenotype in dark grown condition		
			ABA-hypersensitive during seed germination		
*gcr1*	–		Reduces root length		
			Hypersensitivity to ABA-induced inhibition of stomatal opening and promotion stomatal closure, and seed germination		
			ABA-related genes are lightly upregulated		
*gpa1gcr1*	– –		Phenocopies *gpa1*		
*agb1gcr1*	– – –		Phenocopies agb1		
*agb1gcr1gpa1*	– – –		Phenocopies *agb1*		

### Drought stress

**Figure 3:**
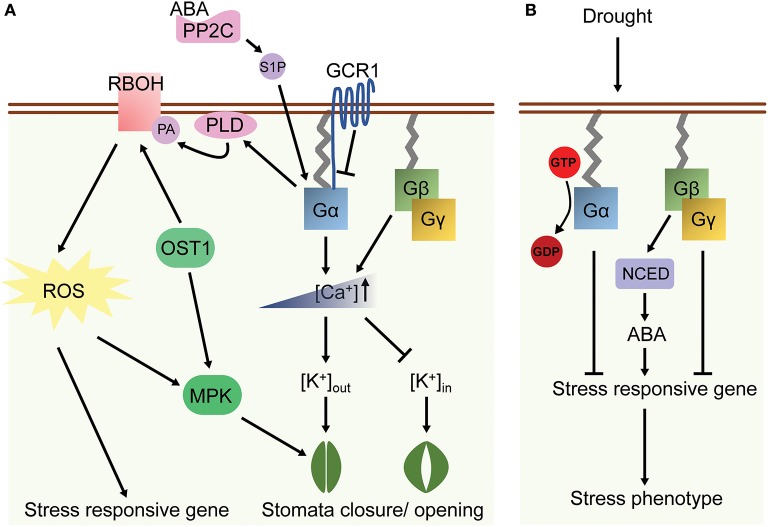
A model of G protein-related ABA and drought responses in plants. **(A)** ABA coupled with PP2C induces Gα activation through Sphingosine-1-phosphate (S1P), leading to the [Ca^2+^] elevation in cytosol. Gβγ also increases the cytosolic concentration of Ca^2+^ under ABA treatment. Followed by changes of K^+^ in- and outflux, the stomatal closure or opening is then regulated by ABA-dependent G protein signaling. In addition, Gα interacts with phospholipase Dα (PLD) and its lipid product phosphatidic acid (PA), which subsequently affects the activity of RBOH. ROS and MAP kinase (MPK) signal are up-regulated correspondingly in ABA response. **(B)** G protein signaling is involved in drought response in rice. Gα and Gγ suppress the expression of stress responsive genes, while Gβ increases their expression through ABA-mediated pathways. The arrows indicate activation and the bars indicate suppression.

Drought decreases soil water content hence increases the concentrations of hydrogen and other ions in soil, which indirectly evokes multiple developmental and physiological changes similar to high salinity responses. Arabidopsis *gpa1* and *gcr1* mutants displayed lower rates of water loss, which resulted in more resistant to drought stress. On the other hand, the *agb1* mutants had a higher rate of water loss under drought treatment due to higher stomata density and hence it was intolerant to drought stress as compared to WT plants (Pandey and Assmann, 2004; Zhang et al., 2008; Nilson and Assmann, 2010b). Phenotypic plasticity is the ability of one genotype to modify phenotypes in response to different environments (Bradshaw, 1965; Huey, 2002). Given the fact that G proteins regulate multiple signaling cross-talk in plants, they were therefore hypothesized to be plasticity genes, in which mutants might affect the degree of plasticity of a trait under environmental changes. Indeed, significant differences in developmental plasticity were observed between WT and G protein mutants for several reproduction-related traits under drought stress. For example, *gpa1* or *gcr1* mutant showed increased plasticity for inflorescence height or fruit number respectively while *agb1* mutants reduced plasticity for inflorescence height, seed number per fruit and total seed production in drought response (Nilson and Assmann, 2010a). Moreover, *agb1* mutants showed increased total seed production under moderate and severe drought stress condition. These data suggested that *AGB1* controls developmental plasticity in response to drought stress. (Table 1 and Figure 3A).

Rice *d1* (rice Gα*-*null mutant, or *rga1*) mutants exhibit a higher photosynthetic rate, root to shoot ratio and greater stomatal conductance under drought stress (Ferrero-Serrano and Assmann, 2016; Ferrero-Serrano et al., 2018). Deletion of a rice Gγ gene is associated with a quantitative trait locus qPE9-1 that enhances drought tolerance including reduced water loss and higher stomatal conductance. In contrast, Gβ RNAi line showed a hypersensitive phenotype to drought which included higher water loss and lower survival rate after drought treatment (Zhang et al., 2015). The transcripts of some stress-related genes were highly upregulated in the Gγ mutant, whereas expression of ABA synthesis genes and qPE9-1 expression are mis-regulated in Gβ mutant under drought stress (Zhang et al., 2015). These observations suggest that Gγ is a negative regulator while Gβ promotes the tolerance of drought response through the ABA-dependent pathway (Table 2 and Figure 3B). Seed-specific overexpression of *AGG3* also improved drought stress tolerance in *Camelina sativa* (Roy Choudhury et al., 2014).

**Table 2:** Response of G protein mutants to environmental stresses and ABA in plants other than Arabidopsis.

**Mutant**	**Species**	**Tolerance**	**Conditions**	**Phenotype**	**OMICS**	**References**
**SALT**						
d1 (*rga1*, DK22)	Rice	+++	0–150 mM NaCl	Higher fresh weight	NA	Misra et al., 2007; Urano et al., 2014; Swain et al., 2017
			Liquid culture	Reduces leaf senescence		
ct2	maize	+++	0–200 mM NaCl	Higher fresh weight		
			Liquid culture	Reduces leaf senescence		
				Cell division was not suppressed		
BnGA1	Brassica napus	?	Up to 200 mM NaCl	Up-regulated		
			Hoagland solution			
BnGB1	Brassica napus	?	Up to 200 mM NaCl	Up-regulated		
			Hoagland solution			
**DROUGHT**						
d1 (*rga1*, DK22)	Rice	+++	Dry soil	Higher net photosynthesis	NA	Misra et al., 2007; Roy Choudhury et al., 2014; Zhang et al., 2015
			75% water content	Greater stomatal conductance		
				Lower leaf temperatures		
RGB1	Rice	– – –	Dry soil	Higher water loss rate		Ferrero-Serrano and Assmann, 2016; Ferrero-Serrano et al., 2018
				Higher stomatal conductance		
				Higher transpiration rate		
				Lower survival rate		
				Lower expression level of stress-inducible genes		
qPE9-1	Rice	– – –	Dry soil	Higher water loss rate		
				Higher stomatal conductance		
				Lower survival rate		
				Lower expression level of stress-inducible genes		
BnGA1	Brassica napus	?	Up to 20% PEG	Up-regulated		
			Hoagland solution			
BnGB1	Brassica napus	?	Up to 20% PEG	Up-regulated		
			Hoagland solution			
35s::AGG3	Camelina sativa	+++	Dry soil	Lower water loss rate		
			60% water content	Higher survival rate		
**ABA**						
SIGGB	Tomato	+++	10–50 μM ABA Agar plate	Reduces sensitivity to ABA during seed germination WT response to ABA in postgermination development and lateral root production	Transcriptome	Alvarez et al., 2015; Zhang et al., 2015; Subramaniam et al., 2016
35s::AGG3	Camelina sativa	+++	0–25 μM ABA	Higher seed germination	Proteome	
				Longer primary and lateral roots		
				Promotion of stomata closure		
RGB1	Rice	– – –	0–80 μM ABA	Lower germination rate	NA	
			Hydroponics	Higher root length reduction		
				Positively regulates ABA-inducible genes		
qPE9-1	Rice	+++	0–80 uM ABA	Higher germination rate		
			Hydroponics	Lower root length reduction		
				Negatively regulate ABA-inducible genes		
**TEMPERATURE**						
d1 (*rga1*, DK22)	Rice	– – –	4°C soil grown	Lower survival rate	NA	Misra et al., 2007; Ma et al., 2015b
BnGA1	Brassica napus	?	4° or 40°C in growth chamber	Down-regulated		
BnGB1	Brassica napus	?	4° or 40°C in growth chamber	Down-regulated		

### Stress hormone ABA

The phytohormone ABA mediates some of the drought and salt stress responses altered by G protein mutations (Lee and Luan, 2012). In guard cells, ABA decreases the influx of potassium ions and reduces the turgor pressure of guard cells, which causes stomatal closure and suppresses light-induced stomatal opening. Arabidopsis *gpa1* mutants had decreased sensitivity to the ABA inhibition of stomatal opening and lacked ABA inhibition of inward K^+^ channels and pH-independent ABA activation of anion channels (Wang et al., 2001). The *gcr1* mutant showed hypersensitivity to ABA-induced and sphingosine-1-phosphate (S1P)-induced inhibition of stomatal opening and promotion of stomatal closure, suggesting that GCR1 and GPA1 have an opposite effect in ABA signaling in guard cells (Pandey and Assmann, 2004; Pandey et al., 2006). Besides the regulation of K^+^ inward channel, ABA induces the opening of Ca^2+^ channel in guard cells. The ABA-induced Ca^2+^-channel opening was disrupted in the *gpa1* mutants, which led to reduced ROS production in response to ABA (Zhang et al., 2011). Nonetheless, *gpa1* mutant showed WT-response to H_2_O_2_ inhibition of stomatal opening and promotion of stomatal closure, indicating that GPA1 regulates ABA reception and ROS production and consequently in the impairment of Ca^2+^-channel activation (Figure 3A). In contrast, *agg1, agg2*, and *agg1,2* double mutants all exhibited WT responses to ABA in stomatal movement in the guard cells. However, *agg3* mutants showed hypersensitivity to ABA inhibition of stomatal opening and the inward K^+^-channel, which phenocopied *agb1* phenotypes in response to ABA (Chakravorty et al., 2011). These observations suggested that Gβγ dimer are required for the ABA signaling in plants.

RGS1 and PLDα1 accelerate the GTPase activity of GPA1, and both RGS1 and GPA1 inhibit the phospholipase activity of PLDα1 (Chen et al., 2003; Zhao and Wang, 2004). Interestingly, phosphatidic acid (PA), a second messenger derived from the lipid-hydrolyzing activity of PLDα1, binds and inhibits the activity of RGS1 (Roy Choudhury and Pandey, 2017), forming a feedback regulatory loop among G protein complex, PLDα1 and PA. RGS1 serves as a positive regulator of ABA-mediated inhibition of seed germination, while PLDα1 plays a role in ABA-regulated stomatal responses in a G protein-dependent manner (Table 1 and Figure 3A) (Chen and Jones, 2004; Zhao and Wang, 2004; Mishra et al., 2006; Fan et al., 2008). Further, *gpa1rgs1* and *gpa1pld*α*1* mutants showed the same phenotype as *gpa1*, whereas *pld*α*1rgs1* mutants behaved similar to *pld*α*1* and WT in ABA-inhibition of seed germination, suggesting that a combinational and complex interaction between PLDα1 and RGS1 with GPA1 regulate ABA response in plants (Roy Choudhury and Pandey, 2016).

### Ozone stress

Long-term exposure to ozone (O_3_) suppresses plant growth and reduces net photosynthesis, which is considered to cause the reduction of crop yield by 5–15% annually (Ludwikow and Sadowski, 2008). Ozone enters plants during the gas exchange through stomata, degrades to reactive oxygen species in extracellular space, and causes foliar bronzing, irregular lesions and bleaching. In addition to the chlorotic and necrotic symptoms, acute ozone treatment in Arabidopsis results in abnormal morphology such as dwarfism and leaf curling. Genetic evidence suggested that G proteins transduce extracellular O_3_ signals to intracellular signaling molecules (Booker et al., 2004; Joo et al., 2005). Arabidopsis *gpa1* and *gpa1agb1* mutants did not display curling leaf phenotype after O_3_ treatments (Table 1). *gpa1* mutation also reduced ion leakage and cell death caused by O_3_-induced oxidative bursts, while *agb1* mutant oppositely displayed hypersensitivity and intolerance to O_3_ treatment. Differential response to ROS production in G protein mutants, mainly *gpa1* and *agb1*, could explain the molecular mechanism underlying the different sensitivities to ozone (Booker et al., 2004). O_3_ causes a biphasic production of ROS occurring from early and late time points. AtrbohD and AtrbohF, membrane-associated NADPH oxidases, trigger the initial ROS signal in adjacent cells, while the late ROS signal was related to tissue damage-associated components. The early and late responses both disappeared in *gpa1* mutants while only the early response was undetectable in *agb1* mutants (Joo et al., 2005). These suggested that GPA1 and AGB1 are both required for the initial ROS signaling in plants, and GPA1 is responsible for the following intercellular signaling and cell death. A transcriptome analysis with various concentrations of O_3_ revealed that the transcripts of *GPA1, AGB1*, and *RGS1* genes were transiently induced by O_3_. However, most of the gene expression changes were similar among WT, *gpa1, agb1*, and *gpa1agb1* double mutants. Further studies beyond transcriptional regulation are required to bridge the gap between physiological changes and molecular mechanisms underlying G proteins regulatory signaling in response to O_3_ (Booker et al., 2012).

### UVB stress and high light

**Figure 4:**
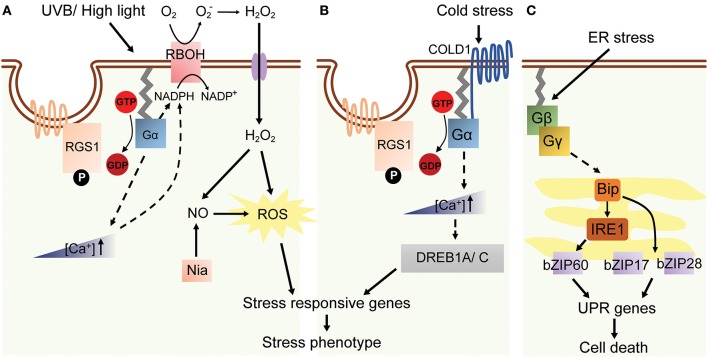
A model of G protein-related high light, cold and ER stress in plants. **(A)** AtGPA1, labeled as Gα in the figure, is activated by high light or UVB stress in Arabidopsis. AtGPA1 induces a transient increase of [Ca^2+^] in cytosol and RBOH activation and the following NO-dependent ROS accumulation. The downstream stress response genes are subsequently up-regulated, which eventually cause the stress phenotype. **(B)** Cold stress response through G protein pathway. COLD1 interacts with AtGPA1 and elevates cytosolic [Ca^2+^] concentration at low temperature. The expression of stress-related TF *DREB1A/C* is upregulated, which leads to the up-regulation of stress responsive genes (Ma et al., 2015a). **(C)** ER stress induces the expression of Gβγ-regulated *Bip, IRE1*, several *bZIPs* and UPR genes.

Depletion of the O_3_ layer has increased the level of high energy UVB radiation, harming most living organisms (Frohnmeyer, 2003). G proteins in mammalian and plant cells are involved in UVB signal transduction (Seo et al., 2004, 2007). In guard cells, UVB radiation induces H_2_O_2_ and nitrogen oxide (NO) generation that causes stomatal closure (He et al., 2013). The UVB-induced stomatal closure did not occur in Arabidopsis *gpa1* mutant, suggesting Gα as a positive regulator of guard cell response to UVB radiation. GPA1 acts as an upstream modulator of H_2_O_2_ and NO, because *gpa1* mutant generates significantly lower levels of H_2_O_2_ and NO under UVB treatment and does not alter stomatal closure induced by these small molecule mediators. The genetic evidence combined with *atrbohD/atrbohF* (defect in H_2_O_2_ production) mutants and *nia1-2/nia2-5* (defect in NO production) mutants, Gα is further confirmed to act as an upstream positive regulator of H_2_O_2_-dependent NO production in in UVB induced stomata closure (He et al., 2013; Figure 4A).

Likewise, plants grown in natural environment are usually exposed to high light (HL) condition and consequently absorb more light energy than required for photosynthesis, therefore the excess excitation energy has to be dissipated (Baker, 2008). In Arabidopsis, high-light stress causes H_2_O_2_ accumulation and induces *ASCORBATE PEROXIDATSE 2* (*APX2*) gene expression. The expression of *APX2* gene was 3–5 fold higher in *gpa1* and *agb1* mutants as compared to WT when grown under high light (Galvez-Valdivieso et al., 2009). Also, HL responsive genes including *HSP17.6C-C1, HSP17.6B-C1*, lipocalin, and *RD20* all showed a similar expression pattern as *APX2* in *gpa1* and *agb1* mutants (Galvez-Valdivieso et al., 2009). These suggested that G proteins are a negative regulator to initiate the downstream pathways under HL stress (Figure 4A).

### ER stress

Causes of ER stress are cellular accumulation of misfolded proteins and disruption of secretory protein synthesis and folding in the ER membrane (Howell, 2013). Two main sensors in response to ER stress have been identified, namely ER-membrane associated transcription factors (bZIP17 and bZIP28) and RNA splicing factors (IRE1A/B) (Howell, 2013). Cleaved bZIP28 shuttles into nucleus and induces the expression of UPR-target genes, such as *Immunoglobulin-Binding Protein 3* (*BiP3*), *Protein Disulfide Isomerase* (*PDB*) and several other components comprising a protein-folding machinery in ER. Wang et al. proposed that Arabidopsis *agb1-2* mutation ameliorates leaf senescence under tunicamycin (Tm) treatment, possibly due to reduced expression levels of BiP3 and PDB transcripts (Wang et al., 2007). However, later studies provided contradicting evidence whereby *agb1-1, agb1-2*, and *agb1-3* all exhibited Tm-induced UPR-sensitive phenotype (Chen and Brandizzi, 2012; Cho et al., 2015) and higher BiP3 expression upon tunicamycin treatment. Gβ and IRE1A/1B seemed to mediate UPR independently from each other, since Gβ- and IRE1A/B-associated UPR signaling pathways additively contributed to ER stress sensitivity (Chen and Brandizzi, 2012). Likewise, *agg1,2,3* and *xlg1,2,3* null mutants both showed similar Tm-induced phenotype, suggesting that Gβγ and XLGs were involved in UPR signaling pathway (Chakravorty et al., 2015; Urano et al., 2016a). The contradicting results from two studies might be due to the unresolved gene network of ER response, therefore requiring further studies to understand the comprehensive role of G proteins in ER stress (Figure 4C).

### Temperature stress

Drastic temperature changes occur often in recent years due to the global warming effect, causing irreversible damage to plants in some cases (Ohama et al., 2017). There are limited studies that focused on the G protein signaling under temperature fluctuations. The *agg2* mutant exhibited slightly earlier flowering phenotype in comparison to WT when grown at higher ambient temperature (around 29°C) (Thung et al., 2013). Heterologous overexpression of *P. sativum* Gα or Gβ in tobacco plants resulted in tolerance to higher temperature during seed germination in T0 and T1 generation transformants (Misra et al., 2007). Heat Shock Factor A1 (HSFA1) is a master regulator in heat stress that initiates the transcriptional cascade of Heat Shock Proteins (HSPs) and other genes for the thermotolerance (Liu et al., 2011; Yoshida et al., 2011). In addition, heat-induced Ca^2+^ and ROS fluctuation may play a role in activation of HSFA1s in response to high temperature (Mittler et al., 2012). On the other hand, warmer ambient temperature sensing in plants requires PHYTOCHROME-INTERACTING FACTOR 4 (PIF4) transcription factor and changes in H2A.Z chromatin status (Kumar et al., 2012). While these transcriptional and epigenetic regulations have been well documented, how the central heat-sensing mechanisms are linked to G proteins-involved heat stress has yet to be investigated in detail.

In cold response, a quantitative trait locus (QTL) protein COLD1 interacts with RGA1 and slightly accelerates its GTPase activity to activate Ca^2+^ channels for conferring chilling tolerance in rice (Ma et al., 2015b). Given the fact that Ca^2+^ signaling mediates cellular responses to altered temperatures and *cold1-1* and *d1* mutants showed a comparable dwarf phenotype which is related to mis-regulated BR and GA pathways, it would be interesting to investigate the molecular network linking hormone, cold response and G protein signals (Figure 4B).

## Systems biology approach to plant G protein research in abiotic stress

Studies on single gene or protein are insufficient to reveal the correlation or the crosstalk between different stress signaling pathways as stress response is a dynamic rather than a static cellular process within a single organism and across different organisms. Moreover, abiotic stress responses in plants involve multiple signaling processes coordinately. Omics studies have the highest potential to uncover the spatiotemporal signaling events for plant stress responses. Various systems biology approaches, for example, transcriptomics, metabolomics, proteomics and interactome have been employed to understand the G protein signaling in abiotic stress responses. These systematic methodologies allow us to not only discover new gene functions in a complex cellular environment, but also characterize genome-scale relationships between genotypes and phenotypes deeply. In this chapter, we discuss established and cutting-edge techniques of OMICs approaches which are applicable to G protein studies.

### Transcriptome

Manipulating massive amounts of transcriptomic data help us systematically grasp co-regulatory modes of gene expressions by G protein pathways, environmental changes and potentially the involvement of phytohormones. In Arabidopsis, *gpa1* and *gcr1* mutations dramatically alter the expression pattern of abiotic stress-related genes, mainly genes related to transcription factors, secondary metabolism and hormone responses (Chakraborty et al., 2015a,b). Likewise, many stress-related genes were mis-regulated in the rice Gα-null mutant *d1* (Jangam et al., 2016; Ferrero-Serrano et al., 2018). Besides these transcriptomic researches, Pandey et al. utilized microarray to comprehensively collect gene expression profiles in G protein mutants treated with or without ABA, then a theoretical Boolean framework was applied to categorize the regulatory modes of each of gene expression changes by *gpa1* or *agb1* mutations as well as ABA treatment (Pandey et al., 2010). The Boolean model enumerated 9 possible G-protein and ABA signaling pathways and 142 regulatory modes (Pandey et al., 2010). This approach not only confirmed the classical mechanisms of G protein signaling but also provided new insight into system specificity of G protein signaling in various cell types. Nonetheless, since gene expression patterns are generally more complex than having only two states (0, 1), data discretization sometimes leads to information loss. In addition, time-series gene expression makes large scale Boolean networks applications difficult. Therefore, it is necessary to collect even more data and apply various methods for modeling gene expression data.

### Metabolome

Despite the intensive researches on physiological changes in G protein mutants under various stresses, information on global profiling of metabolites in G protein mutants in stress condition has remained limited. Metabolomics is a high-throughput approach to enable quantitative and comprehensive identification of large numbers of small metabolic compounds and their dynamic changes to extracellular stimuli. A time-course metabolomics in *gpa1* and WT guard cells identified 85 metabolic compounds and their dynamic changes after ABA treatment. ABA treatment significantly altered 56 and 43 out of 85 metabolites in WT and *gpa1* guard cells, respectively (Jin et al., 2013). Among them, different temporal modules have been found in *gpa1* vs. WT, including Ca^2+^ and other hormone signaling pathways, suggesting ABA serves as an upstream signal to trigger G protein signaling (Jin et al., 2013).

### Proteome

Abundance of transcripts often does not reflect changes in protein abundance. Since proteins are the final products of most genes, proteomes permit a thorough understanding of G protein signaling induced by different stress environments. Few studies have focused on changes in total protein abundance in roots, guard cells and seeds of Arabidopsis Gα mutants and WT (Zhao et al., 2010; Alvarez et al., 2011). iTRAQ, a quantitative proteome approach, identified dozens of polypeptides of which abundance is cooperatively changed by ABA and G protein signaling in guard cells and roots. Novel G protein functions such as ER body formation and intracellular trafficking may shed light on new roles of Gα in Arabidopsis. These proteins include proteins related to intracellular trafficking in roots and to photosynthesis in guard cells. These two studies revealed how tissue specificity is involved in different G protein functions in plants (Zhao et al., 2010; Alvarez et al., 2011). The seed proteome from overexpressed *AtAGG3* transgenic *Camelina sativa* has been investigated by liquid chromatography (LC)-based quantitative proteomics approach (Alvarez et al., 2015). In addition to proteins which are associated to hormone regulation, seed size and drought tolerance consistent with its physiological observation, proteins related to heavy metal responses have been identified, suggesting the involvement of AGG3 in heavy metal tolerance (Alvarez et al., 2015). However, limited total number of proteins (around 1,500–2,000 proteins in each report) were found in these studies, most of which are abundant proteins in the plastids, which might result in loss of information and bias toward data interpretation. Methods to achieve higher sensitivity and accuracy, such as optimal enrichment, fractionation and protein digestion protocols, could be employed for increasing the coverage of low abundant proteins.

### Interactome

Proteomics approaches have been developed to identify not only protein abundance related to G protein signaling, but also interacting partners of G protein subunits. Two yeast-two-hybrid (Y2H) based interactome studies identified hundreds of G protein-interacting partners (Klopffleisch et al., 2011; Jones et al., 2014). Among them, Klopffleisch et al. (2011) comprehensively screened for interacting partners of Arabidopsis G protein subunits, and a following study has found that salt stress-related proteins as an overrepresented group (Colaneri et al., 2014). A Y2H experiment was carried out for identifying the binding partners of XLGs. Seventy-two potential proteins were found to interact with XLG1, 2, and 3 and more than 70% of them were confirmed to bind with XLGs *in vivo* using BiFC. The results not only provided new insight into XLG's newly identified binding partners which participated in G-proteins mediated salt response, but also provided valuable stress-related protein set for further studies. However, the detailed mechanism still remains unclear (Liang et al., 2017). Y2H method only detects the direct interactions between bait and prey proteins, although plausible indirect interactions can be deduced from *in silico* network construction. Moreover, protein-protein interactions are a dynamic process and sometimes the post-translational modification such as phosphorylation or ubiquitination is critical for the interaction. Hence, an immunoprecipitation (IP)-MS based interactome was established to detect the time- and glucose- dependent RGS1 interacting networks (Jaiswal et al., 2016). One hundred nineteen proteins were identified as RGS1 interactors, among which 93 were novel targets associated with transport, stress and metabolism at low glucose levels, and vascular trafficking and signal transduction at high glucose levels, respectively (Jaiswal et al., 2016). More recently, Yu et al. (2018) utilized co-IP and liquid chromatography (LC)-MS to isolate and identify AGB1-assoicated proteins. A total of 103 candidate AGB1-associated proteins were identified including all of the G protein subunits except XLG1, receptor-like kinase, Ca^2+^ signaling-related proteins and 14-3-3-like proteins. Among them, FER was confirmed to physically interact with AGB1 by using BiFC and was involved in ABA-regulated stomata opening and closure in a G-protein dependent manner. However, the AGB1-assoicated proteins in the control condition did not differ from those identified in the salt treatment condition, suggesting that the AGB1-dependent salt response signaling was likely involved in the more downstream pathways (Yu et al., 2018).

## Novel approaches to study abiotic stress response- time-course or cell-type specific network construction/ PTM/ CHIP-SEQ TF network

The above-mentioned systematic approaches greatly improved our knowledge in plant abiotic stress responses and the involvement of G protein pathways, however the application of these advanced approaches is still limited to specific cases such as signals evoked by ABA or some stress treatments. With the importance and complexity of G proteins-by-environment relationship, more profound and broader studies are required. This section describes novel approaches that have yet to be applied to plant G protein signaling.

### High resolution spatiotemporal gene regulatory network to reveal multiple phases in response to abiotic stress

Given the rapid development of OMICs tools, an increasing number of researches are utilizing these techniques to construct comprehensive visualization of stress responses in plants. These techniques enable detection and quantification of dynamic intracellular changes from gene expressions to post-translational modifications of proteins during the course of stress response and development. For example, Geng et al captured spatiotemporal transcriptional changes in different cell types during Arabidopsis root development under salt treatment (Geng et al., 2013). Their high-resolution transcriptional map demonstrated that ABA signaling pathways spatially regulate salt stress-specific transcriptional programs in selected layers of Arabidopsis root tissue to promote growth recovery from high salinity. In contrast, sodium toxicity independently regulates many tissue- and time-specific transcriptional responses which are associated with water transport and hydrophobic cell wall tissue (Casparian strip) formation. By combining highly resolved time series transcriptome and a dynamic modeling, an integrative visualization of the temporal response to drought in Arabidopsis can be achieved. Notably, Bayesian network modeling of TF genes was applied to infer the differentially expressed gene regulatory networks that mediate the transition from the early to late stage of drought response (Bechtold et al., 2016). This approach has predicted that Agamous-Like 22 (AGL22) is a key hub in this regulatory network and the follow-up genetic studies confirmed that AGL22 regulates the transcriptional network during drought stress, linking changes in primary metabolism to the initiation of drought response. As the large amounts of transcriptomic dataset are available publicly, meta-analysis has emerged to aim for compelling the results across independent studies and extract the most robust and useful information. A study has employed meta-analysis and meta-regression to normalize public transcriptomic dataset from Arabidopsis in response to water loss (Rest et al., 2016). This novel approach identified the genes with small differential responses consistently in all the analyzed dataset, which contributed to stress tolerance.

### ChIP-sequencing to identify whole genome TFs network and epigenetic regulations in abiotic stress condition

ChIP-sequencing identifies binding targets of multiple TFs and their directly regulatory genes. Through integrated-TF networks of ABA-induced drought response, Song et al identified 21 ABA-related TFs and novel genome-wide binding sites in Arabidopsis (Song et al., 2016). An extensive feedback of ABA regulatory network was predicted by analyzing chronological changes of differentially expressed genes and differentially binding motifs under ABA treatment. Based on the prediction, multi-TF binding could be a criterion for prioritizing the further characterization of unknown genes with genetic methods in plants. Several novel TFs that are involved in ABA and salt regulatory signaling were uncovered with the following proof-of-concept experiments.

In addition, ChIP-sequencing is capable of detecting chromatin dynamics and variations. Environmental factors induce epigenetic changes such as chromatin modification, which are tightly correlated with transcriptional response. For example, drought, flooding, temperature fluctuation and high salinity affect the methylation or acetylation of DNA and histones (Pandey et al., 2016; Asensi-Fabado et al., 2017). A genome-wide survey of methylation status in response to salt stress in rice revealed that the level of hypo-methylation is associated with the expression level of DNA demethylases, and this led to different degrees of salt tolerance in two contrasting rice lines (Ferreira et al., 2015). It was also found that histone deacetylase HDA6 is crucial for H3K4me3-mediated gene activation, changing the sensitivity toward salt stress in Arabidopsis (Sani et al., 2013). Moreover, the expression of drought stress responsive genes showed a positive correlation with the level of histone modifications H3K9ac and H3K4me3 during the course of drought treatment, and the drought treatment resulted in genome-wide variations in H3K4me1, H3K4me2 and H3K4me3 in Arabidopsis (van Dijk et al., 2010; Kim et al., 2012). Another whole genome association study focusing on temperature response revealed that the variation of DNA methylation patterns was strongly associated with genetic variations and their growing temperatures (Dubin et al., 2015). Further researches are required to investigate if G protein pathways regulate these epigenetic changes and how the epigenetic changes are related to transcriptional changes and physiological outcomes in G protein mutants.

### Post-translational modification (PTM)-phosphoproteome

PTM, particularly phosphorylation, participates in the signal transduction in abiotic stress conditions. Several quantitative phosphoproteomes of drought stressed plants (Umezawa et al., 2004, 2013; Wang et al., 2013) revealed that SUCROSE NON-FERMENTING 1-RELATED KINASES 2 (SNRK2) family transmits ABA-induced signals through phosphorylation of downstream substrates. By comparing the phosphoproteome of *snrk2* mutants and WT, Umezawa et al identified new direct substrates of ABA-activated SNRK2 (Umezawa et al., 2013). Similarly, a phosphoproteome research in crops in response to drought has identified phosphorylation events of some common stress-related proteins such as Ca^2+^ signal-related proteins and HSPs (Rampitsch and Bykova, 2012). A time-course phosphoproteome from rice roots under salt stress showed that the phosphorylated stress-responsive proteins are differentially expressed with prolonged salt stress. Several novel membrane proteins including aquaporins and photosystem II-related proteins were phosphorylated in response to salt condition (Chitteti and Peng, 2007). In summary, proteomics and PTM identification could shed light on uncovering new protein targets that are involved in stress tolerance as well as identifying novel phosphorylation events within signal transduction pathways. G proteins regulate ABA-response genes as well as Ca^2+^ signaling pathways. Future studies should investigate the whole genome PTM status in G protein mutants in comparison to WT under various stresses.

### Deep learning in OMICs researches

As discussed, OMICs data are one of the higher order dimensional data with complex multi-level structures, and therefore they have become promising input data for machine learning and deep learning approaches for analysis and interpretation of biological data. Indeed, machine learning has been implemented in abiotic stress research recently (Ma et al., 2014). A prediction model was built based on the training with public abiotic stresses transcriptomic dataset to recognize common or distinct patterns. The comparative gene expression network was applied to find distinct and novel gene candidates related to abiotic stress response, two mutants of which demonstrated salt hypersensitive phenotype. Moreover, several deep learning techniques such as deep neural networks (DNN), convolutional neural networks (CNN) and recurrent neural networks (RNN) have been applied to biomedical, drug discovery and fundamental biology researches to identify new regulators from integrated big OMICs data. For example, Chen et al (Chen et al., 2016) applied DNN to gene expression data to infer expression levels of 21000 target genes from 1,000 landmark genes and showed highly precise prediction of gene expression model. Furthermore, Simm et al (Simm et al., 2018) applied DNN to reanalyze high-throughput bio-images for predicting the specificity and activity of new drugs, boosting the speed of drug discovery. Likewise, a CNN-based algorithm has been implemented to learn regulatory sequences of quantitative trait loci and disease-associated variants from large-scale chromatin-profiling data, which resulted in better understanding of complex disease-associated SNPs (Zhou and Troyanskaya, 2015). Recently, A RNN-based integrative model has been designed for predicting molecular state in *E. coli*. This model is trained based on multi-omics and interaction data and then predicts multi-omics expression under untested novel conditions. As a result, it could precisely predict and integrate different layers of OMICs data and could be broadly applicable for biological discovery (Kim et al., 2016). Given the fact that increasing numbers of OMICs data from different stress conditions and of easily adapted deep learning libraries are being available, a new possibility will be opened to reanalyze OMICs data and then build a training model based on well-developed algorithms for discovering new regulators associated with G proteins in plants.

## Conclusions and future perspectives

**Figure 5:**
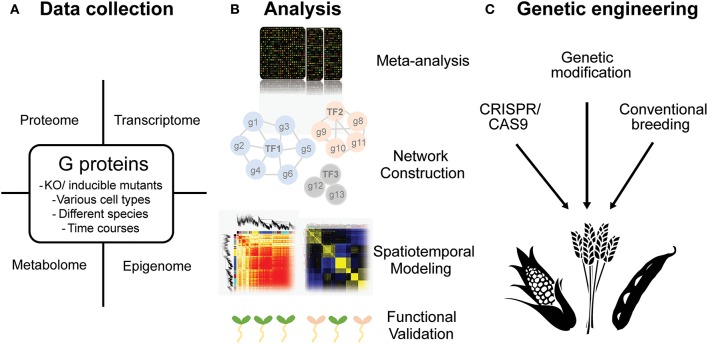
Potential perspectives in the research of G protein regulated stress response in plants. **(A)** Different types of OMICS data such as transcriptome, proteome, epigenome and metabolome could be sampled from various tissues or along with different developmental stages in plants. **(B)** Several systematic-analytical techniques could be taken including meta-analysis, unsupervised and supervised network construction, spatiotemporal modeling and functional validating by phenomes. **(C)** The ultimate goal is to utilize the knowledge obtained from model plants and to create stress tolerant crops by cutting-edge biotechnologies.

Evidence to date leave no doubt that G proteins are involved in abiotic stress response in many plants. However, to access the in-depth knowledge on how G proteins are involved in stress response, more genome-wide analyses are still needed. As discussed, the methodologies and experimental designs in systems biology help us answer the complex questions in abiotic stress responses (Figure 5). We could also implement similar strategies to open questions in G protein science. Examples of these questions are listed below.

### Time resolution

Stress response is by no means a steady-state response and could be well-explained by the changes captured in just a few time points under stress treatment. However, stress responses in plants are extremely dynamic along the time. Consequently, the time resolution of exact kinetic changes in G proteins and their regulated genes after stress signal perception would be an interesting topic in the future.

### Cell type and tissue specificities

Previous studies suggested that G proteins acquired different specificities in different cell types in response to ABA. Plants also show different responses to various abiotic stresses in different cell types. Hence, it would be interesting to elucidate G protein regulatory network in distinct cell types in different developmental stages under stress condition. Besides, there are several public databases regarding gene expression patterns in different root or seed cell types along the course of development. By comparing gene expressions changed by abiotic stresses and G proteins using the publicly-available data, we might gain insight into the common and distinct regulatory modules associated with G protein signaling under stress conditions.

### Beyond transcriptional regulation

**Figure 6:**
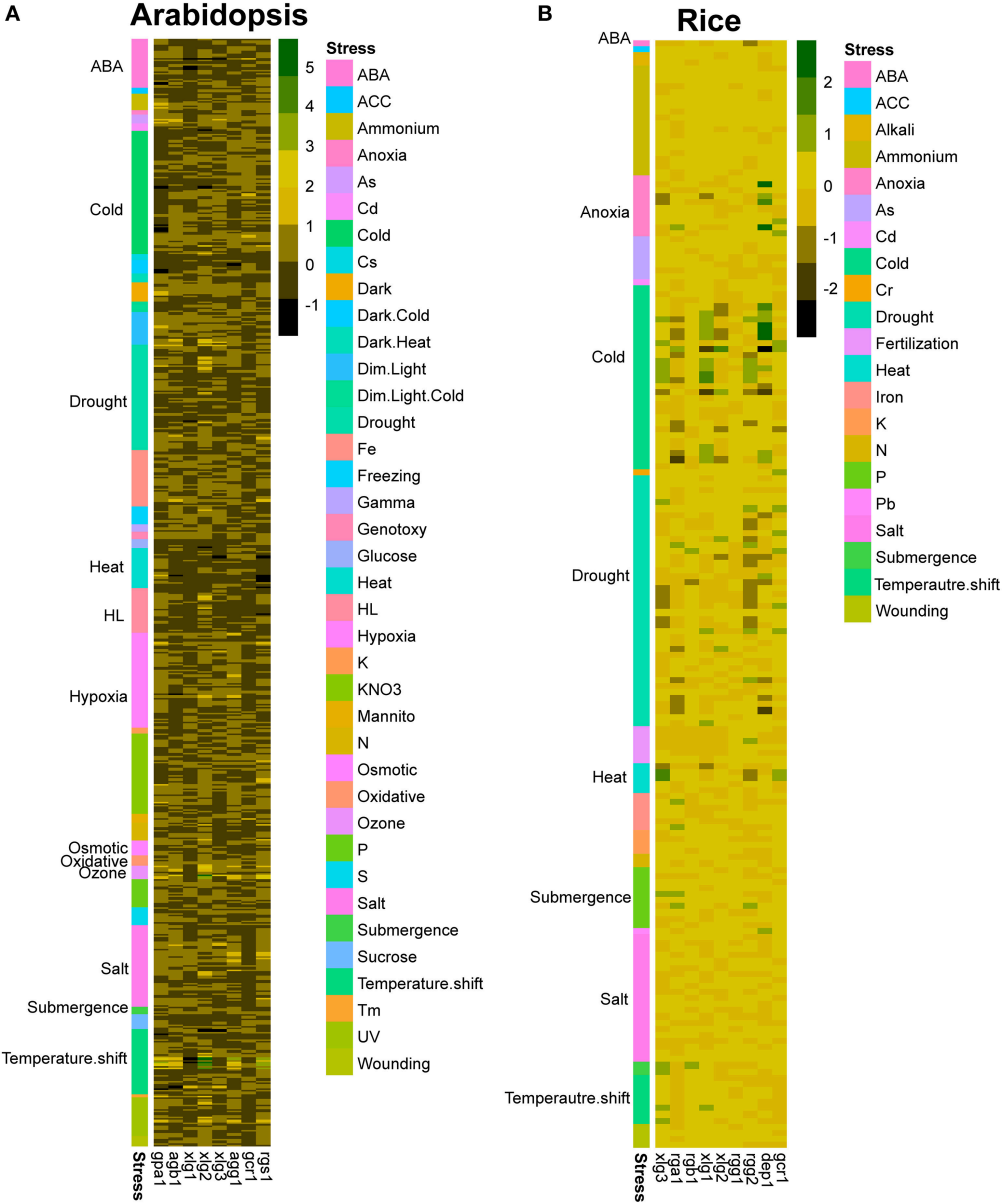
Expression patterns of G protein-related genes in response to various abiotic stresses. Expression profiles of G protein-related genes in **(A)** Arabidopsis and **(B)** rice under various stress conditions including chemical treatments, hormone treatments, various light intensity and quality, various temperature treatments, osmotic stress and drought stress are obtained from GENEVESIGATOR. The expression data were log_2_ transformed, and *p*-values were calculated by the comparison between each treatment and the control condition. Data with *p* < 0.05 was selected.

By analyzing the expression patterns of G protein genes from public data, it is clear that the expression levels of G protein genes are relatively consistent, only exhibiting some slight changes under certain conditions (Figure 6). While molecular mechanisms for stress-related phenotypes in G protein mutants are unclear yet, it is evident that G proteins regulate downstream proteins through the changes of PTM status. In addition, PTMs particularly phosphorylation would regulate the activity of G proteins themselves. Therefore, investigating the genome-wide PTM status is particularly important to understand the signaling flow and activation state of G protein complex.

### Mechanisms to integrate various stresses

It has been a long-standing question how plants sense different stress conditions and activate different stress response genes. Abiotic stress responses are mediated by several signaling pathways: some are shared by different stresses while others are unique to a specific stress type. G proteins are involved in many stress tolerance, but little is known about their selectivity. A possible strategy adopted by plants would be switching interacting partners based on the type of stresses. More specifically, G proteins may form a protein complex with different regulatory partners upon different types of stresses. Interactome or proteome experiments could be established to determine the protein components associated with G protein complex under different stress conditions.

### Stress response mechanisms conserved across species

To date the evidence show that part of the G protein response to stress is similar in Arabidopsis and several crops including rice and maize (Urano et al., 2014; Ma et al., 2015b; Zhang et al., 2015; Ferrero-Serrano and Assmann, 2016). However, phylogenetic analyses indicated that some G protein components were lacked or duplicated in different species, therefore it would be interesting to investigate if G protein-related stress responses remain conserved. Systems biology strategies would also shed light on how plants evolve to tolerate different and constantly changing environmental stresses.

## Conclusion

In conclusion, with the development of OMICs tools and integration of massive amounts of quantitative dataset, we are able to understand the molecular and biochemical aspects of G protein regulation in various environmental stresses. The long-term goal is to integrate the outcomes from previous conventional studies and future genome-wide studies to find new stress tolerance gene candidates and mechanisms in plants. The new knowledge will help us to genetically design crops which are able to flourish in changing environment.

## Author contributions

T-YW and DU contributed to the conception, design and writing of the manuscript.

### Conflict of interest statement

The authors declare that the research was conducted in the absence of any commercial or financial relationships that could be construed as a potential conflict of interest.
